# Subtotal versus total parathyroidectomy: retrospective patient-centric outcomes in a chronic dialysis population

**DOI:** 10.1186/s12882-025-04335-5

**Published:** 2025-07-16

**Authors:** Raymond Lin, Mirna Vucak-Dzumhur, Eva Wong, Hsiang Chung, Grahame J. Elder

**Affiliations:** 1 Faculty of Medicine and Health, University of Sydney, Sydney, NSW Australia; 2 Department of Renal Medicine, Nepean Hospital, Sydney, NSW Australia; 3 Department of Renal Medicine, Westmead Hospital, Westmead, NSW Australia; 4 School of Medicine, University of Notre Dame Australia, Sydney, NSW Australia; 5 School of Medicine, Western Sydney University, Sydney, NSW Australia; 6 Department of General Surgery, Westmead Hospital, Westmead, NSW Australia; 7 Department of General Surgery, Nepean Hospital, Kingswood, NSW Australia

**Keywords:** Subtotal parathyroidectomy, Total parathyroidectomy, Auto-transplantation, Hyperparathyroidism, Dialysis, Bone mineral density

## Abstract

**Background:**

Hyperparathyroidism occurs commonly in the dialysis population, and surgical parathyroidectomy (PTx) is often required when medical therapy to suppress parathyroid hormone (PTH) fails. Surgical techniques include subtotal and total PTx, with or without auto-transplantation, with the choice of procedure generally determined by surgical preference rather than patient-related factors. The aim of this study was to compare outcomes of these surgical procedures, focusing on post-operative utilization of hospital resources, and biochemical and patient-level outcomes over the year following surgery.

**Methods:**

This retrospective observational study included dialysis patients undergoing subtotal or total PTx (± auto-transplant) over 9-years at three tertiary-level hospitals in Sydney, Australia. Laboratory and patient-level-outcomes were compared immediately post-operatively and at one, three and 12-months.

**Results:**

Of 64 dialysis patients undergoing PTx, 60.9% were male and the mean dialysis vintage was 5.9 (4.2) years. Total PTx was performed in 51, 46 with auto-transplantation, and subtotal PTx in 13. Patient characteristics were similar at baseline. Compared to subtotal PTx, total PTx resulted in lower post-operative calcium values (*p* = 0.01), higher intravenous calcium requirements (*p* = 0.03) and more frequent admission to intensive care (*p* = 0.03). After total PTx, the daily calcium and calcitriol pill burden at discharge was higher (median 25 (IQR 20–40) vs. 18 (IQR 6–26), *p* = 0.04) and at 3-months (*p* = 0.01), and 23.5% of patients were readmitted for calcium management (*p* = 0.05). At 12-months, more patients undergoing subtotal PTx had PTH values above guideline recommendations (42.9% vs. 9.3%, *p* = 0.02), pill burdens did not differ, and bone mineral density increased in both groups.

**Conclusions:**

Total PTx requires greater post-operative resources but is associated with lower PTH values at 12-months, whereas subtotal PTx is associated with a lower pill burden but increased hyperparathyroidism recurrence. A tailored strategy is suggested, matching the surgical approach to patient needs.

## Background

Hyperparathyroidism is a commonly diagnosed component of the chronic kidney disease-mineral and bone disorder (CKD-MBD) in patients on dialysis. CKD progression is frequently accompanied by disturbances in calcium and phosphate homeostasis, vitamin D metabolism, klotho expression and fibroblast growth factor-23 production. These changes may lead to initially adaptive and homeostatic, but ultimately maladaptive changes in parathyroid hormone (PTH) production [1], characterized by continuous stimulation of PTH release and parathyroid gland hyperplasia. An important consequence of severe hyperparathyroidism is a mismatch between bone formation and resorption, resulting in reduced bone mass, increased bone fragility, and a high propensity for fracture [1, 2]. Medical management of severe hyperparathyroidism with phosphate binders, vitamin D analogues and calcimimetics can achieve some suppression of PTH secretion, however, many patients require surgical parathyroidectomy (PTx) for definitive management.

Current Kidney Disease Improving Global Outcomes (KDIGO) guidelines suggest PTx in patients who fail to respond to medical or pharmacological therapy [3]. PTx can be classified as total (removal of all glands) or subtotal (removal of less than all glands), with or without auto-transplantation of resected glands [4]. Whilst both approaches achieve an acceptable reduction in PTH, there is no consensus on the optimal approach, with centre-to centre variations usually based upon surgical preference and expertise. Patient characteristics, such as age and kidney transplant candidacy might also influence surgical decision-making.

The main benefit of total PTx, usually with auto-transplantation, appears to be reduced rates of recurrence, which may be advantageous in older people who become unsuitable for re-operation over time [5, 6]. Some studies have noted an increase in cardiovascular events following total PTx [7], but this is not a consistent observation [8]. Others have demonstrated non-inferiority of the subtotal approach compared to total PTx, with a lower risk of long-term hypocalcaemia [9]. Prolonged relative hypoparathyroidism may result in high calcium and active vitamin D requirements, a high pill burden, and potentially low or adynamic bone turnover, which could potentiate vascular calcification [10].

Many studies comparing subtotal and total PTx have focused on PTH reduction, safety and long-term recurrence [11, 12], but few have compared immediate post-operative outcomes of hypocalcaemia, intravenous (IV) calcium use, intensive care unit (ICU) admission and lengths of stay, as well as longer-term medication burden. The aim of this study was to compare outcomes of these surgical procedures, focusing on post-operative utilization of hospital resources, and biochemical and patient-level outcomes over the year following surgery.

## Methods

### Study design and population

This was a retrospective observational study of all dialysis patients > 18-years-old, undergoing PTx in the Western-Sydney Renal Service (WRS) network over 9 years from 1^st^ January 2015 to 31^st^ December 2023. The WRS network comprises three metropolitan, tertiary-level hospitals in Western Sydney, New South Wales, Australia.

Patients were identified from electronic hospital medical records using the Australian Classification of Health Intervention (ACHI) code for ‘Subtotal Parathyroidectomy’ and ‘Total Parathyroidectomy’. The surgical procedures of all identified patients were confirmed by accessing the procedural logbooks of surgeons who performed PTx within the network. Dialysis patients undergoing parathyroid surgery were identified by cross-referencing with the Renal Information System Catalogue (RISC), a database of all dialysis and transplant patients within the network.

### Databases

Baseline characteristics, patient medications, pre- and post-operative parameters and outcome data were obtained through electronic medical records shared across the three hospitals. Dialysis-related information and mortality data were obtained through RISC.

### Baseline characteristics

The following demographic and baseline information were collected: age (at time of PTx), gender, dialysis modality (haemodialysis (HD), peritoneal dialysis (PD), both), dialysis vintage, history of fracture, history of calciphylaxis and number of hospital admissions in the 12-months preceding PTx. Patient medications at the time of PTx were documented, including calcitriol (the only funded vitamin D receptor agonist available in Australia), cinacalcet (the only funded calcimimetic), calcium and non-calcium-based phosphate binders, oral and intravenous bisphosphonates, denosumab and corticosteroids.

### Pre-operative parameters

The following pre-operative biochemical parameters were obtained: corrected calcium, magnesium, phosphate, PTH, alkaline phosphatase (ALP), 25-hydroxyvitamin D and 1,25-dihydroxyvitamin D. If preoperative values were missing, values were accessed up to three months before surgery.

### Subtotal versus total parathyroidectomy determination

PTx operation reports and corresponding histopathology reports were collated for each patient. De-identified reports were evaluated by a head and neck surgeon as to a) the number of parathyroid glands identified during the operation, b) the number of glands resected, c) whether an autotransplant was performed. Procedures were classified as total PTx if all identified glands were removed with or without autotransplant, and subtotal PTx if less than all identified glands were removed, with or without autotransplant.

### Post-operative outcomes

Immediate post-operative outcomes of interest were: lowest measured post-operative serum corrected calcium, use of IV calcium replacement, ICU admission, ICU length-of-stay (LOS), total hospital LOS, discharge calcitriol dose/day (mcg) and calcium dose/day (mg).

At 1-month, 3-months and 12-months, serum corrected calcium, phosphate, PTH, ALP, calcitriol dose (mcg/day) and calcium dose (mg/day) were recorded. Readmissions to hospital post-discharge were documented and categorized as: a) readmissions for hyper- or hypocalcaemia, b) readmissions for cardiovascular-related causes, c) readmissions for vascular access-related causes, d) all other readmissions. Mortality was recorded.

### Data analysis

Normally distributed continuous variables are presented as mean and standard deviation (SD), skewed distributions are presented as median and interquartile range (IQR) and categorical variables are presented as number (%). Independent continuous variables were analyzed by T-test and linear regression with correlated outcomes by paired T-test. Continuous variables with skewed distributions were analyzed by Wilcoxon Rank Sum (Mann-Whitney U) test. Independent binary variables were analyzed by Chi-square test and logistic regression with correlated outcomes using McNemar’s chi-square test. Statistical analyses were performed with Stata version 13.0 (Stata, AU). Patients who received a kidney transplant were censored from the time of transplant. Mortality analysis excluded patients receiving a kidney transplant.

## Results

### Population and surgery characteristics

During the study, 64 patients on chronic dialysis underwent subtotal PTx (*n* = 13) or total PTx (*n* = 51), with no significant differences at baseline. The majority of total PTx patients had autotransplantation (*n* = 46, 90.2%), versus two patients undergoing subtotal PTx (15.4%). Table 1 includes baseline characteristics and pre-operative laboratory data for the total cohort, and for those undergoing total and subtotal PTx. The majority of patients (95%) received pre-operative calcitriol loading with no differences between total and subtotal PTx groups (*p* = 0.17). Eleven patients (seven total PTx, four subtotal PTx) received a kidney transplant, ranging from 10 days to 11 months post-PTx.

**Table 1:** Baseline characteristics and pre-operative biochemistry. There were no significant differences in any variable between the surgical groups

	All (n = 64)	Subtotal PTx (n = 13)	Total PTx (n = 51)
***Baseline characteristics***			
Age (years) (SD)	48.2 (16.5)	55.8 (20.0)	46.3 (15.1)
Gender (male) (%)	39 (60.9)	8 (61.5)	31 (60.8)
Dialysis modality (n) (%)			
*Haemodialysis*	27 (42.2)	6 (46.2)	21 (41.2)
*Peritoneal dialysis*	14 (21.8)	2 (15.4)	12 (23.5)
*Both modalities*	23 (35.9)	5 (38.5)	18 (35.3)
Dialysis duration (years) (SD)	5.9 (4.2)	4.7 (2.9)	6.2 (4.4)
Previous transplant (n) (%)	11 (17.2)	1 (7.7)	10 (19.6)
Fracture history (n) (%)	21 (32.8)	3 (23.1)	18 (35.3)
Calciphylaxis (n) (%)	2 (3.1)	0	2 (3.9)
Medications (n) (%)			
*Calcitriol*	25 (40.3)	5 (41.7)	20 (40.0)
*Calcium-based phosphate binder*	20 (31.8)	5 (38.5)	15 (30)
*Non-calcium-based phosphate binder*	54 (87.1)	10 (83.3)	44 (88.0)
*Oral bisphosphonate*	17 (27.4)	1 (8.3)	16 (32.0)
*IV bisphosphonate*	3 (4.8)	1 (8.3)	2 (4.0)
*Denosumab*	0	0	0
*Cinacalcet*	26 (42.6)	4 (33.3)	22 (44.9)
*Corticosteroids*	3 (4.8)	0	3 (6.0)
***Pre-operative Biochemistry***			
Corrected calcium (mmol/L) (SD)	2.48 (0.21)	2.46 (0.21)	2.48 (0.21)
Magnesium (mmol/L) (median, IQR)	0.98 (0.87–1.10)	0.88 (0.79–1.01)	1.00 (0.87–1.09)
Phosphate (mmol/L) (SD)	1.81 (0.5)	1.83 (0.54)	1.84 (0.55)
Parathyroid hormone (pmol/L) (median, IQR)	175.9 (123.9–243.6)	150 (128.3–205.3)	196.4 (122.9–252.2)
Alkaline phosphatase (U/L) (median, IQR)	317 (163–683)	193 (144–452)	386 (168–694)
25-hydroxyvitamin D (nmol/L) (SD)	62.6 (32.5)	58.3 (27.6)	63.3 (33.6)
1,25-dihydroxyvitamin D (pmol/L) (median, IQR)	30 [15-43]	48 (40–56)	29 (15-37)

### Subtotal versus total parathyroidectomy outcomes

#### Immediate post-operative outcomes

Post-operatively, patients undergoing total PTx differed from those undergoing subtotal PTx, by having a lower serum calcium nadir (1.99 mmol/L vs. 2.19 mmol/L, *p* = 0.01) and higher post-operative intravenous calcium requirements (75% vs. 42%, *p* = 0.03), with intravenous calcium given when serum corrected calcium values fell below 2.00 mmol/L. Patients undergoing total PTx had higher rates of ICU admission (96% vs. 75%, *p* = 0.03), but no differences in ICU LOS or hospital LOS (Table 2). At discharge, patients undergoing total PTx had higher prescribed calcium doses and pill counts for calcitriol and calcium (25/day vs. 18/day, *p* = 0.04), and both groups were discharged on high doses of calcitriol, which did not differ significantly) (Table 2).

**Table 2:** Immediate and subsequent post-operative outcomes

	Subtotal PTx (n = 13)	Total PTx (n = 51)	*p*-value
**Immediate Post-operative Outcomes**			
Post-operative lowest serum corrected calcium (mmol/L) (SD)	2.19 (0.21)	1.99 (0.22)	0.01*
Patients requiring post-operative IV calcium (n) (%)	5 (41.7)	36 (75)	0.03*
ICU admission rate (n) (%)	9 (75)	43 (95.6)	0.03*
ICU length-of-stay (days) **†** (median, IQR)	3 (2–4)	3.5 (2–6)	0.26
Total hospital length-of-stay (days) (median, IQR)	5 (3–10.5)	5 (4–10)	0.76
Discharge calcitriol dose (mcg/day) (median, IQR)	3 (1–4)	4 (4–6)	0.07
Discharge calcium dose (g/day) (median, IQR)	2.4 (1.8–3.6)	5.4 (3.6–7.2)	0.01*
Discharge total pill count** (n) (median, IQR)	18 (6–26)	25 (20–40)	0.04*
**Subsequent Post-operative Outcomes**			
1-month: Corrected calcium (mmol/L) (SD)	2.36 (0.07)	2.36 (0.04)	0.95
Alkaline phosphatase (U/L) (median, IQR)	166 (117–439)	382 (162–599)	0.09
Calcitriol dose (mcg/day) (median, IQR)	1 (0.5–2)	2 (0.8–4)	0.12
Calcium dose (g/day) (median, IQR)	1.2 (0.6–1.2)	2.7 (1.2–5.1)	0.02*
Total pill count** (n) (median, IQR)	6 (3–10)	14 (6.5–26)	0.06
3-month: Corrected calcium (mmol/L) (SD)	2.30 (0.07)	2.38 (0.05)	0.42
Alkaline phosphatase (U/L) (median, IQR)	110 (73–193)	113 (86–193)	0.81
Parathyroid hormone (pmol/L) (median, IQR)	33.4 (1.6–60.5)	7.1 (1–17.6)	0.13
Calcitriol dose (mcg/day) (median, IQR)	0.5 (0.1–1.5)	1 (0.5–2)	0.04*
Calcium dose (g/day) (median, IQR)	0.6 (0–1.2)	2.1 (1.2–3.6)	0.01*
Total pill count** (n) (median, IQR)	3 (0.4–8)	9 (5–12)	0.01*
12-month: Corrected calcium (mmol/L) (SD)	2.22 (0.08)	2.33 (0.03)	0.23
Alkaline phosphatase (U/L) (median, IQR)	120 (63–166)	80 (63–125)	0.28
Parathyroid hormone (pmol/L) (median, IQR)	34.0 (9.2–115.4)	6.6 (2.3–24.3)	0.02*
Calcitriol dose (mcg/day) (median, IQR)	0.5 (0.1–1)	0.5 (0.25–1)	0.68
Calcium dose (g/day) (median, IQR)	0.6 (0–1.8)	0.6 (0–1.2)	0.88
Total pill count** (n) (median, IQR)	3 (0.4–6)	3 (2–7)	0.70
Readmissions for hyper- or hypocalcaemia (n) (%)	0	12 (23.5)	0.05*
Parathyroid hormone < 1.7pmol/L (n) (%)	0	10 (19.6)	0.08

ICU = intensive care unit, IQR = interquartile range, IV = intravenous, PTx = parathyroidectomy, SD = standard deviation. **†** ICU length-of-stays only calculated for those with an ICU admission

**Pill count refers to prescribed calcitriol (0.25 mcg) and calcium carbonate (600 mg) tablets

### Subsequent post-operative outcomes

**Fig. 1:**
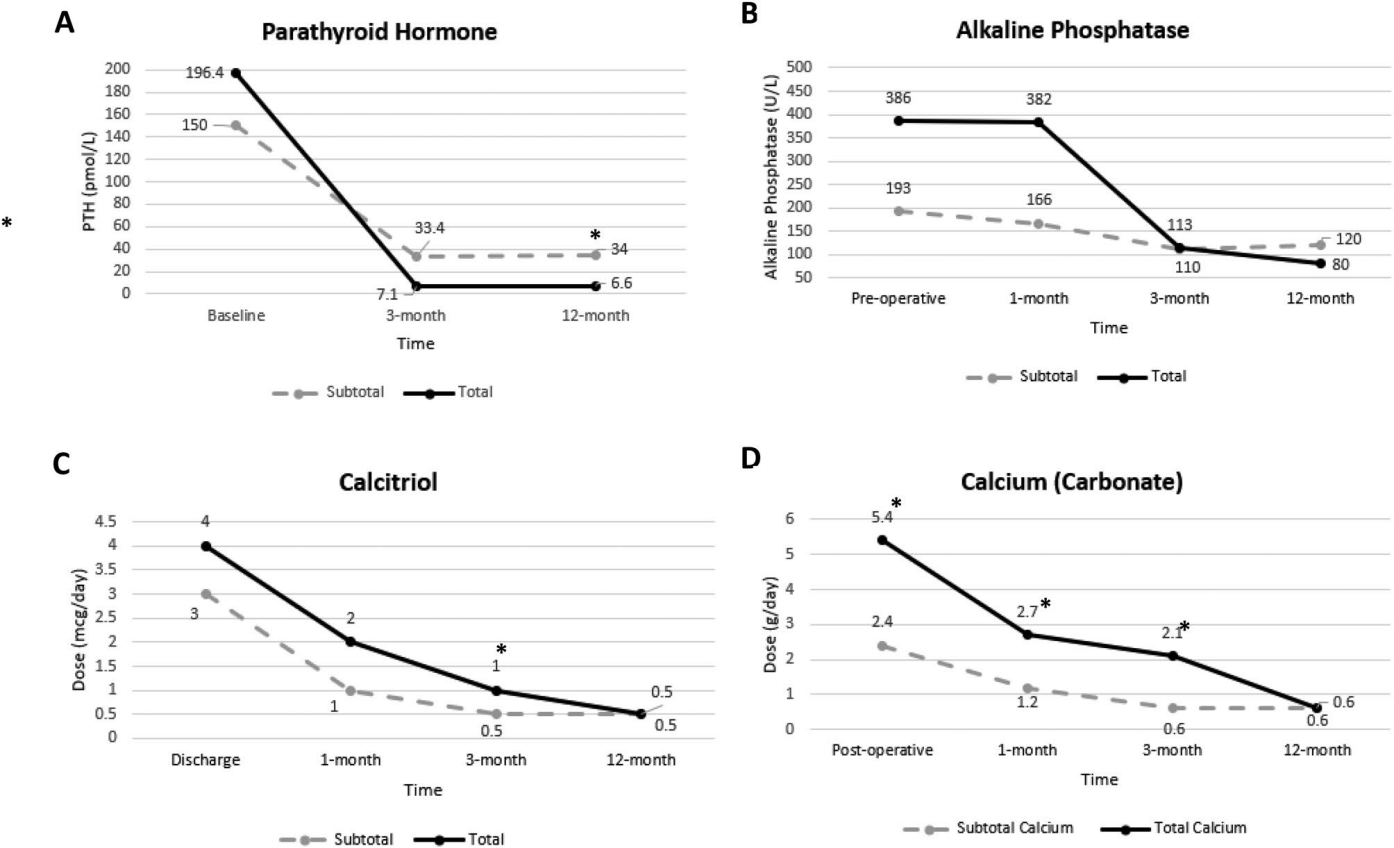
Post-operative PTH, ALP and pill burden. (**A**) Parathyroid hormone levels (median) at baseline, 3-months and 12-months. (**B**) Alkaline phosphatase (median) at baseline, 1, 3 and 12-months. (**C**) Calcitriol dosage (mcg/day) (median) at discharge, 1-month, 3-months and 12-months. (**D**) Calcium carbonate dosage (mg/day) (median) at discharge, 1-month, 3-months and 12-month

At 1-month, patients undergoing total PTx had a higher daily calcium dose (2.7 g/day vs. 1.2 g/day, *p* = 0.02), and at 3-months total PTx patients had higher daily doses of calcitriol (1.0mcg/day vs. 0.5mcg/day, *p* = 0.04), calcium (2.1 g/day vs. 0.6 g/day, *p* = 0.01) and the total pill burden (9/day vs. 3/day, *p* = 0.01). There were no significant between-group differences in serum calcium and ALP at 1- and 3-months. At 12-months, median PTH values differed between total and subtotal PTx patients (6.6 pmol/L (IQR 2.3–24.3) vs. 34.0 pmol/L (IQR 9.2–103.0), *p* = 0.02) and a higher proportion of patients undergoing subtotal PTx had PTH values > 9-fold the upper normal range of the PTH assay (42.9% vs. 9.3%, *p* = 0.02). Pill burden was similar between groups at 12-months. Figure 1 depicts trends in PTH, ALP, calcitriol and calcium doses over time. At 12-months, 10 patients (19.6%) in the total PTx group had PTH values < 1.7pmol/L (the lower normal range of the assay) (Table 2). Nine of these 10 patients had undergone auto-transplantation.

### Post-parathyroidectomy hospital admissions

In the total PTx group, 12 patients (23.5%), all of whom had an autotransplant, were readmitted for management of hyper- or hypocalcaemia vs. no subtotal PTx patients (*p* = 0.05) (Table 2).

The percentage of patients admitted for at least one cardiovascular event in the 12-month periods pre- and post-PTx was 20.3% vs. 20.3%, with no differences between those undergoing total and subtotal PTx (*p* = 0.65). For patients undergoing PTx, admission rates for vascular access dysfunction were higher in the 12-months after PTx than in the 12-month prior to PTx (48% vs. 36%, *p* = 0.01) with no between-group differences.

### Mortality

Within 12-months of surgery, two patients who underwent subtotal PTx died from cardiac arrest and bowel ischaemia.

**Table 3:** Pre- and post-operative changes in DXA-derived bone mineral density, T-scores and Z-scores

**Dual Energy X-ray Absorptiometry**			
**Site**	**BMD and DXA Parameters**	**T-Score: mean (SD)**	**Z-Score: mean (SD)**
**Pre-operative BMD (n = 21)**			
Lumbar spine (g/cm^2^) (SD)	1.061 (0.256)	−1.2 (2.1)	−0.9 (2.0)
Total hip (g/cm^2^) (SD)	0.834 (0.170)	−1.9 (1.3)	−1.5 (1.3)
Femoral neck (g/cm^2^) (SD)	0.833 (0.188)	−1.7 (1.4)	−1.2 (1.4)
Distal 33% radius (g/cm^2^) (SD)	0.667 (0.144)	−3.0 (1.4)	−2.8 (1.5)
Trabecular bone score (L1-4)	1.328 (0.156)		
**Post-operative change in BMD (n = 10)**			
	**Bone density change** **(g/cm**^**2**^)**(SD)**	**Percentage BMD Change (SD)**	***p*** **-value**
Lumbar Spine	+0.231 (0.228)	+23.5 (26.0)	0.01
Total hip	+0.148 (0.136)	+20.1 (19.4)	0.01
Femoral neck	+0.143 (0.151)	+20.3 (21.8)	0.02
Distal 33% radius	+0.048 (0.016–0.043) (IQR)	+9.5 (−0.3–7.7) (IQR)	0.18

BMD = bone mineral density, DXA = dual-energy X-ray absorptiometry, IQR = interquartile range, SD = standard deviation

### Associations of hypocalcaemia and hospital length of stay

Post-operative hypocalcaemia was associated with higher pre-operative ALP (*p* = 0.001), with total PTx (*p* = 0.03) and with female gender (*p* = 0.04), but there were no significant associations with age, dialysis vintage, pre-operative serum calcium, phosphate, PTH, history of fracture, use of anti-resorptive medications or cinacalcet use. Baseline variables were not associated with ICU LOS, but higher preoperative ALP values were associated with longer hospital LOS (*p* = 0.04).

## Bone mineral density changes

Pre-operative dual-energy X-ray absorptiometry (DXA) studies were available for 21 patients measured using a General Electric Lunar iDXA fan beam bone densitometer. Scans were performed on average 5.8 ± 4.4 months pre PTx. Baseline bone mineral density (BMD), T-scores and Z-scores are summarized in Table 3. At baseline, mean T-scores at the lumbar spine, total hip and femoral neck were in the osteopenic range and T-scores at the distal 1/3 radius were in the osteoporotic range. Baseline Z-scores, corrected for age and sex, did not differ between total and subtotal PTx groups.

Of the 21 patients with pre-operative DXAs, 10 had a further DXA 5 to 24-months post-operatively (2 subtotal and 8 total PTx). Changes from the baseline BMD (g/cm^2^ and percent) are summarized in Table 3. BMD increased significantly at the lumbar spine, total hip and femoral neck. Subtotal and total PTx groups were not analyzed separately.

## Discussion

In this study, immediate post-operative resource usage was higher in patients undergoing total PTx, who experienced a lower calcium nadir and higher requirements for post-operative IV calcium (75% vs. 41.7%). Intravenous calcium was given when serum corrected calcium values fell below 2.00 mmol/L. Its use requires careful monitoring, and extravasation from intravenous sites can induce tissue necrosis. Assuming successful total PTx, even with auto-transplantation, there will generally be a period of hypoparathyroidism before autotransplanted glands successfully engraft [13]. During this period, the sudden reduction in PTH stimulation of bone resorption and increased maturation of inchoate osteoblasts can lead to unopposed mineral apposition resulting in hypocalcaemia, known as the hungry bone syndrome (HBS). This may persist for weeks to months. Conversely, after subtotal PTx, at least one parathyroid gland is left wholly or partially undisturbed, conferring a lower risk of hypocalcaemia. The association of increased post-operative hypocalcaemia with total versus subtotal PTx has been reported infrequently [9, 14], possibly due to variations in monitoring and calcium-replacement protocols.

The overall rate of ICU admission in this study was high at 91.2%, because the local PTx protocol recommends serum calcium monitoring in an ICU setting unless the patient is deemed low-risk. Despite high ICU usage, there were fewer admissions following subtotal than total PTx, with a trend toward shorter ICU LOS. Total hospital LOS was similar, with a median of 5-days in both groups, and the finding of preoperative ALP as the main association of LOS is concordant with other studies [15].

At hospital discharge, supplemental calcium doses after total PTx compared to subtotal PTx, were significantly higher, with median doses of 5.4 g/day vs. 2.4 g/day respectively, and a trend to higher calcitriol doses. Considering calcium carbonate is given as 600 mg tablets and calcitriol as 0.25mcg capsules, there was an appreciable difference in pill burden, with total PTx patients taking a median of 25 pills/day at discharge vs. 18 pills/day for subtotal PTx patients (*p* = 0.04). The difference in pill burden was seen to three months, with total PTx patients requiring an additional 8 and 6 pills/day at one and three months respectively.

For patients with already high medication requirements, this increased medication burden could predispose to non-adherence and error [16]. Although we were unable to quantify compliance, high pill burdens increase the risk of non-adherence and may be a consideration in some patients. Due to higher calcium requirements, patients with total PTx may also have more labile post-operative serum calcium values, with a reduced ability to compensate for under-replacement, or an increased propensity to ‘overshoot’ calcium targets. In keeping with this, patients undergoing total PTx in this study had higher rates of readmission for management of hyper- or hypocalcaemia (23.5%) vs. no readmissions in the subtotal PTx group (*p* = 0.05). All these patients had autotransplants, so differences may be attributable to the delay before effective engraftment, or to differences in pill requirements and possibly compliance. The available but limited literature comparing readmission rates following total and subtotal PTx is conflicting, with some investigators finding no differences and others reporting total PTx as an independent risk factor [17, 18].

A clinically important difference between groups was the PTH value at 12-months, with a median of 34.0 pmol/L following subtotal PTx and 6.6 pmol/L after total PTx. Zmijewski et al. in a retrospective study of 46 dialysis patients, found PTH values > 21 pmol/L were more frequent at ≥6 month from subtotal PTx [9]. Of note, 42.9% of subtotal PTx patients had PTH values greater than KDIGO recommended targets by 12-months [3]. Two recent systematic reviews and meta-analyses found no differences in rates of hyperparathyroidism recurrence between total and subtotal PTx groups [11, 12], but these studies included dialysis and renal transplantation populations, and their follow-up periods were relatively short at six months, or highly varied [11, 12]. Combining dialysis and transplant patients is contentious, because patients on dialysis remain exposed to disturbances of calcium and phosphate homeostasis that predispose to hyperparathyroidism. Additionally, carboxyl-terminal PTH fragments contribute around 50% of the measured intact PTH in patients on dialysis [19], whereas these non-calcaemic fragments are cleared following transplantation. Finally, parathyroid hyperplasia progresses over time, so longer follow-up periods are preferable. Our data suggests subtotal PTx may be more suitable for patients likely to receive kidney transplantation in the short to medium term before recurrent hyperparathyroidism becomes an issue. Conversely, total PTx may be more appropriate for patients who are less suitable for possible reoperation, such as older patients with greater comorbidities.

A potential risk accompanying lower 12-month PTH values is relative hypoparathyroidism. In this study, ten patients undergoing total PTx (nine having had autotransplantation) had PTH values < 1.7pmol/L at 12 months, versus no patients undergoing subtotal PTx. These observations are consistent with other reports of an increased risk of persistent hypoparathyroidism after total PTx [9, 20]. Nevertheless, 12-month ALP values did not differ significantly, consistent with preserved bone turnover in both groups. Patients with hypoparathyroidism may be at increased cardiovascular risk [21] and, compared to subtotal PTx, increased cardiovascular events have been reported after total PTx [7]. However, as hyperparathyroidism itself predisposes to cardiovascular disease and mortality [22], discriminating between risks and benefits of total PTx is complex. Studies have reported stabilization or improvement in coronary and other vascular calcifications post-PTx [23, 24], however patients with hypoparathyroidism and low bone turnover probably represent a distinct subpopulation. Hernandez et al. performed bone biopsies in 19 HD patients before and after PTx, reporting an association between vascular calcification progression and the severity of low bone turnover [10]. Although there was no increase in cardiovascular admissions post-operatively this study was not designed to include cardiovascular confounders. We did observe an increase in admissions for vascular access dysfunction in the year following versus the year prior to PTx, but this may reflect increasing dialysis vintage. Post-PTx vascular access dysfunction has been reported previously [25], so this outcome merits consideration in future studies.

Finally, although post-PTx changes in bone mineral density were only assessed in 10 patients, we observed significant increases of approximately 20% at the lumbar spine, total hip and femoral neck, consistent with other reports [26, 27]. Few studies have compared bone mineral density outcomes following total and subtotal PTx, and numbers in this study precluded that analysis. It is unclear whether sometimes extreme BMD gains during periods of positive calcium balance [28] accelerate vascular calcification.

This study has several strengths, with data from three tertiary hospitals over a 9-year period, and care provided by different surgeons and physicians, reducing centre bias. As the hospitals share electronic medical records and a dialysis database, baseline and follow-up laboratory data were accessible on all patients, reducing risks of sampling bias. The study is one of only few assessing medication-burden at differing time points, and the 12-month follow-up period allowed for the detection of divergence in PTH values.

This study also has limitations. A retrospective observational study design is not as robust as randomized controlled studies, however randomization to total or subtotal PTx is generally not feasible, with choice primarily dictated by surgical preference, and pre- and intraoperative factors. Moreover, differences between total and subtotal PTx outcomes will be influenced by remnant gland size, auto-transplantation technique and patient anatomy. Although cardiovascular and vascular access-related hospital admissions were documented, the study had limited numbers and was not designed to correct for potential confounders.

## Conclusions

Despite being more resource-intensive in the first months post-operatively, total PTx appears to provide better sustained remission of hyperparathyroidism, albeit with a possible increase in the risk of hypoparathyroidism. However, the potential for increased post-operative management needs consideration and discussion with patients or carers. Subtotal PTx is associated with lower post-operative resource use, a lower pill burden and less readmissions for calcium management, but requires greater vigilance to avoid recurrent hyperparathyroidism, and may be better suited for kidney transplant candidates rather than patients remaining on long term dialysis. The current study suggests a tailored surgical approach to PTx based on patient factors. Further analysis of the pooled outcomes of these surgical approaches is needed to inform and shift peri-operative decision-making toward patient-centric outcomes.

## Data Availability

De-identified data supporting the conclusions of this article will be made available by the authors on reasonable request.

## Abbreviations

ACHI: Australian Classification of Health Intervention
ALP: Alkaline phosphatase
AU: Australia
BMD: Bone mineral density
CKD: Bone mineral density
CKD-MBD: Chronic kidney disease mineral bone disorder
DXA: Dual-energy X-ray absorptiometry
HD: Haemodialysis
ICU: Intensive care unit
IQR: Interquartile range
IV: Intravenous
KDIGO: Kidney Disease Improve Global Outcomes
LOS: Length of stay
PD: Peritoneal dialysis
PTH: Parathyroid hormone
PTx: Parathyroidectomy
SD: Standard deviation
RISC: Renal Information System Catalogue
WRS: Western Renal Service
